# Cytotoxicity of poly-guanidine in medulloblastoma cell lines

**DOI:** 10.1007/s10637-023-01386-z

**Published:** 2023-08-09

**Authors:** Gabriel Gallo-Oller, Teresita Díaz de Ståhl, Ayodele Alaiya, Sten Nilsson, Anders R. Holmberg, Marcela Márquez-Méndez

**Affiliations:** 1 Childhood Cancer Research Unit, Department of Women’s and Children’s Health, Karolinska Institutet, Stockholm, Sweden; 2 Department of Oncology-Pathology, Karolinska Institutet, Stockholm, Sweden; 3 Cell Therapy and Immunobiology Department, King Faisal Specialist Hospital and Research Centre Oncology Centre, Riyadh, Saudi Arabia; 4 Center for Research and Development in Health Sciences, Autonomous University of Nuevo León, Monterrey, N.L. Mexico

**Keywords:** Poly-guanidine, Polyamines, DAOY, MB-LU-181, Sialic acid, Cytoskeleton polymerization, DNA condensation, Cytotoxicity efficacy, Cell migration

## Abstract

Medulloblastoma (MB) is the most common pediatric brain tumor. The therapy frequently causes serious side effects, and new selective therapies are needed. MB expresses hyper sialylation, a possible target for selective therapy. The cytotoxic efficacy of a poly guanidine conjugate (GuaDex) incubated with medulloblastoma cell cultures (DAOY and MB-LU-181) was investigated. The cells were incubated with 0.05–8 µM GuaDex from 15 min to 72 h. A fluorometric cytotoxicity assay (FMCA) measured the cytotoxicity. Labeled GuaDex was used to study tumor cell interaction. FITC-label *Sambucus nigra* confirmed high expression of sialic acid (Sia). Immunofluorescence microscopy was used to visualize the cell F-actin and microtubules. The cell interactions were studied by confocal and fluorescence microscopy. Annexin-V assay was used to detect apoptosis. Cell cycle analysis was done by DNA content determination. A wound-healing migration assay determined the effects on the migratory ability of DAOY cells after GuaDex treatment. IC_50_ for GuaDex was 223.4 -281.1 nM. FMCA showed potent growth inhibition on DAOY and MB-LU-181 cells at 5 uM GuaDex after 4 h of incubation. GuaDex treatment induced G2/M phase cell cycle arrest. *S. nigra* FITC-label lectin confirmed high expression of Sia on DAOY medulloblastoma cells. The GuaDex treatment polymerized the cytoskeleton (actin filaments and microtubules) and bound to DNA, inducing condensation. The Annexin V assay results were negative. Cell migration was inhibited at 0.5 µM GuaDex concentration after 24 h of incubation. GuaDex showed potent cytotoxicity and invasion-inhibitory effects on medulloblastoma cells at low micromolar concentrations. GuaDex efficacy was significant and warrants further studies.

**Supplementary Information:**

The online version contains supplementary material available at 10.1007/s10637-023-01386-z.

## Introduction

Medulloblastoma (MB) is a type of brain tumor that develops in the cerebellum at the back of the head, in the posterior fossa. It is the most common malignant pediatric brain tumor, and it is characterized by its high cellularity and fast proliferation [1]. However, it may also occur in younger adults. MB can metastasize to other parts of the brain and spine but seldom spreads to other body parts. The 2021 WHO molecular classification defines MB into four subgroups: Wingless (WNT), Sonic Hedgehog (SHH), Group 3, and Group 4. Each subgroup presents defined characteristics according to gender, age distribution, metastasis, and clinical outcome. Among them, the subgroup SHH-activated, and TP53-mutant is the most aggressive, with the worst prognosis. This subgroup represents approximately 25% of the MB diagnosis [2].

Surgery is standard for managing primary MB, and its role in recurrent MB is still being determined [3]. Gross surgical resection (GTR, gross tumor resection) followed by radiation and risk-adapted cerebrospinal fluid adjuvant chemotherapy have produced the best survival results. The estimated 5-year survival is approximately 70% and ~ 60% for children with metastatic MB or subtotal resection [4].

Recurrence occurs in up to 30% of children and represents the most common cause of death. More than 95% of relapsing patients die. The high treatment failure rate at recurrence raises questions about whether additional surgery or intensive chemotherapy is warranted at this stage of the disease [3]. In addition, secondary malignant neoplasms may develop following therapy [5].

Survivors of MB have an increased risk of experiencing a decreased quality of life owing to the adverse effects of the treatment, which can affect brain development and cause neurologic damage. In addition, the mass effects of MB itself may also cause lasting damage [6, 7].

The actin cytoskeleton is often disrupted in cancer, leading to altered F-actin properties that can contribute to neoplastic transformation, tumor growth, and metastasis [8]. Furthermore, during mitosis, dynamic crosstalk exists between microtubules, actin, and the plasma membrane [9]. Therefore, aberrant actin isoform expression could be used as a potential biomarker for early cancer onset, a potential therapeutic target, and an indicator of the effectiveness of chemotherapeutic agents [10].

Some drugs act on the components of the cytoskeleton. For example, Taxol stabilizes microtubules, and Phalloidin stabilizes actin filaments. Other drugs, such as Cytochalasin D, bind to actin monomers and prevent their polymerization into filaments [11]. Cytochalasin and the toxin Latrunculin prevent actin polymerization and inhibit cell movements such as locomotion. Due to the lack of specificity for the types of actine (i.e., cannot distinguish between cardiac, smooth muscle, muscle, and cytoskeletal forms of actin), the use of these drugs in animal results in unacceptable off-target effects [12].

Microtubules are structural components of the cell that are involved in a wide variety of functions, including cell shape, motility, intracellular trafficking, and mitotic spindle formation. Consequently, microtubules are suitable targets for anticancer agents.

Drugs can bind to tubulin and modify its assembly properties, interfering with microtubule dynamics, which can halt the cell cycle and result in apoptosis. However, data suggest that the interference of microtubule dynamics is insufficient to halt cell mitosis [13]. Previous studies have demonstrated that suppression of microtubule dynamics can occur at concentrations lower than those needed to block mitosis. In addition, suppressing microtubule dynamics by tubulin mutations or drug treatment has been shown to inhibit cell migration [14].

Overexpression of β3-tubulin in clinical samples correlates with tumor aggressiveness, resistance to chemotherapeutic drugs, and poor patient survival [15, 16].

Immunofluorescence studies have shown that DAOY cells, a widely studied model of MB, show low expression of **γ**-tubulin and overexpression of β3-tubulin localized in the mitotic spindle [17]. The detection of βIII-tubulin on DAOY cells in mitotic spindle microtubules has potential implications in cancer chemotherapy, as microtubules enriched in β3-tubulin exhibit increased dynamic instability and chemoresistance to tubulin-binding agents as taxanes [18].

GuaDex is a cationic polydisperse carbohydrate polymer with conjugated guanidine groups with a molecular weight of ~ 55 kD. It belongs to the class of polyamines, which are organic compounds characterized by the presence of two or more amino groups in their structure, which give them their positive charge. The potential of GuaDex as an antitumor agent has been demonstrated in various malignant cell types, including CNS tumors [19–21].

### Mode of action hypothesis

The cationic nature of GuaDex allows it to bind electrostatically to the negatively charged tumor cell membrane and swiftly internalize via the polyamine uptake system. Once inside the cell, Guadex induces toxicity through electrostatic cross-linking with negative charge phosphorylated molecules in the cytoplasm and nucleus (DNA), causing lethal damage [20]. In addition, malignant cells' high metabolic needs are expressed by a polyamine hunger [22, 23] that facilitates GuaDex’s fast internalization.

## Material and methods

### Cell cultures

DAOY desmoplastic MB cell line from SHH group (ATCC-LGC Standards, Middlesex, UK) was cultured in DMEM high glucose supplemented with 10% fetal bovine serum (FBS) (GIBCO, Paisley, UK), 4 mM glutamine (GIBCO, Paisley, UK), and 1% penicillin/streptomycin (GIBCO, Paisley, UK). MB-LU-181 neurospheres group 3 primary tumor with high stem cell content was established by Dr. John Inge Johnsen, Karolinska Institutet. MB-LU-181 is a resistant cell line to cisplatin, temozolomide, and etoposide treatment but showed intermediate sensitivity to the ROCK inhibitors and vincristine [24]. Its phenotype has high proliferation, expression of tumor markers, MYC amplification, and elevated MYC expression. DAOY and MB-LU-181 cell lines characteristics are shown in Table 1. The MB-LU-181 neurospheres were cultured according to Sandén et al. [25]. In brief, cells were seeded on Ultra-LowTM 6-well plates (Corning, VWR, Spånga, Sweden) in UltraCULTURE™ cell culture medium (Lonza BioWhittaker Inc., VWR) supplemented with 2 mM L-glutamine (Lonza BioWhittaker Inc., VWR), 1% antibiotics (Penicillin–Streptomycin, Life Technologies) and 20 ng/ml EGF (Chemicon, Merck Millipore, Solna, Sweden). Following sphere formation, the spheres were mechanically passaged and further propagated. All cultures were maintained at 37 °C in a humidified atmosphere with 5% CO2.

**Table 1:** Human medulloblastoma cell lines

**Cell lines**	**Group**	**Characteristics and expression**	**IC**_**50**_** (μM)**	**References**
**DAOY**	SHH	Adherent TP53 mutated, ROCK2 moderate expression ROCK1, pMLC2, MLC2, GAPDH, and Vinculin are positive.		Dyberg et al. [24]
			ROCK inhibitors RKI-1447 = 10.8 AT13148 = 2.92 HA1077 = 124	
			MYC inhibitor JQ1 = 10	Sandén et al. [25]
		CD133 and cMyc negative, GFAP 75%, Nestin 99%, Sox1 50%, Sox2 40%, ßIII-tubulin 70%, ß-Actin, CD24 90%, CD44 99%, Ki-67 99% are positive.		Casciati et al. [26]
		Tetraploid		Jacobsen et al. [27]
**MB-LU-181**	3	Neurospheres High proliferation. Myc amplified, ROCK1 and Vinculin positive, pMLC2 and MLC2 negative, ROCK2 moderate expression, Resistant to cisplatin, temozolomide and etoposide. Sensible to Vincristine		Dyberg et al. [24]
			ROCK inhibitors RKI-1447 = 4.38 AT13148 = 5.65 HA1077 = 51.5	
		CD133 positive, Myc amplified, cMyc, cyclin B1, nestin, nf-200, Il-8, Il-16, COX-2, and VEGFA have high expression. GFAP, CD15 and CD44 are negative		Sandén et al. [25]
			Myc inhibitor JQ1 = 0.27	

RKI-1447, AT13148 and HA1077; ROCK inhibitors

*SHH* sonic hedgehog, *GFAP* Glial fibrillary acidic protein antibody, *ßIII-tubulin* cytoskeletal protein, *CD133 (promin-1)* antigen glycoprotein cancer stem cell biomarker, *CD24* sialoglycoprotein, *CD44* antigen cell-surface glycoprotein, *CD15 (Sialyl-Lewis*^*x*^*)* tetrasaccharide composed of sialic acid, fucose and an N-acetyllactosamine, *KI-67* proliferation biomarker, *cMyc* transcription factor and proto-oncogene, *Nestin* neuroepithelial stem cell biomarker, *Sox2* transcription factor for pluripotency, *ß-Actin* cytoskeletal protein, *ROCK* Rho-associated protein kinase, *GAPDH* glyceraldehyde-3-phosphate dehydrogenase enzyme, *nf-200* anti-neurofilament 200 antibody, *pMLC2* phosphor-myosin light chain 2 antibody, *Vinculin* actin binding protein, *VEGFA* vascular endothelial growth factor A, *COX-2* cyclooxygenase (prostaglandin-endoperoxide synthase)

The characteristics of DAOY and MB-LU-181 patient-derived MB cell lines are detailed in Table 1.

### Lectin binding detection

A fluorescein-elderberry bark lectin (SNA) (Immunkemi F&D AB, Järfälla, Sweden) was used as sialic acid (SIA) expression control ligand. This lectin binds preferentially to sialic acid linked to galactose in α-2,6, and to a lesser degree, α-2,3 linkage. The cell lines were cultured on chamber slide glass 8-well (Nunc, VWR, Stockholm, Sweden) for 24 h. The cells were rinsed in 0.15 M NaCl + 10 mM HEPES two times. Twenty µg /mL of SNA lectin in PBS buffer was added to the monolayer cells and neurospheres, which were then incubated for 2 h in the dark at room temperature (RT). After the incubation, the cells were rinsed with NaCl-HEPES buffer three times. Finally, the cells were fixed for 15 min in 70% ethanol and mounted in a mounting medium with DAPI (Vectashield, Immunkemi F&D AB, Järfälla, Sweden). A confocal microscope (Leica SP5, with a Leica Application Suite advance fluorescence 2011 Software, Leica Microsystems GmbH, Wetzlar, Germany) was used for image analysis.

### GuaDex binding analysis

GuaDex was prepared as described previously [28]. Briefly, Dextran 70 PhEUR (Pharmacosmos AS, Denmark) was oxidized, and aminoguanidine (Sigma-Aldrich, St. Louis, MO, USA) was subsequently conjugated with reductive amination. Sodium borohydride (Sigma-Aldrich, St. Louis, MO, USA) was used for the reduction. Disposable PD-10 desalting Sephadex G-25 columns were used for purification (Cytiva Sweden AB, Uppsala, Sweden).

Fluorescein isothiocyanate (FITC) GuaDex labeling was prepared as described previously [19]. Briefly, 300 μg FITC solution (Sigma-Aldrich, St. Louis MO, USA) was mixed with 1 mL of GuaDex (15 mg/mL), in 0.02 M borate buffer at pH 9,5. The solution was incubated on a shaker overnight in the dark and at RT. After incubation, the solution was purified on a PD-10 column equilibrated with PBS. Rhodamine-labeling of GuaDex was prepared as the previous labeling method but with 500 μg rhodamine B isothiocyanate (Sigma-Aldrich, St Louis MO. USA) mixed with 1 mL of GuaDex (15 mg/mL) in 0.02 M borate buffer at pH 9,5 as described previously [21]. Cells were cultured on chamber slide glass 8-wells for 24 h. FITC-GuaDex or Rhodamine-GuaDex at 5 μM concentration in 500 μl PBS buffer were added to the cells. After incubating (1–4 h in the dark at RT), the cells were rinsed with PBS three times and fixed for 15 min in 70% ethanol. Then, the cells were prepared with a DAPI mounting medium (Vectashield, Immunkemi F & D AB, Järfalla, Sweden). A confocal microscope (Leica SP5) and a fluorescent microscope (Axioplan 2, Zeiss) were used for the image analysis.

### Fluorimetric cytotoxicity assay (FMCA)

Cytotoxicity was determined with a fluorometric cytotoxicity assay (FMCA), as described by Larsson and Nygren [29]. Briefly, ~ 8,000 cells were seeded in 96-well plates (Falcon; Becton Dickinson, Meylan, France). DAOY cells were incubated for 24 h to allow attachment and MB-LU-181 for 48 h to obtain spheres. Then cells were incubated in 5 µM GuaDex at 1 min to 4 h, and 0.5 µM to 8 µM concentrations incubated for 72 h. The control wells were furnished with PBS. All the experiments were done with six-duplicate wells. Plates were centrifuged, and cells were washed twice with DPBS/Modified (HyClone™ Laboratories, Utah, USA). The cells were washed in PBS. Fluorescein diacetate (FDA) (Sigma-Aldrich, St. Louis MO, USA) was dissolved in DMSO and kept frozen at -20 °C as a stock solution (10 mg/mL). The FDA was diluted in PBS at 10 μg/mL, and 200 μl was added to each well. Then, the plates were incubated for 30 min at 37 °C. A 96-well scanning fluorometer (Infinite^®^ 1000Pro-Tecan, Grödig/Salzburg, Austria) was employed, and the data was analyzed by Magellan Software (version 6.6) (Tecan Trading AG, Grödig/Salzburg, Austria). The results were calculated using Microsoft Excel. Data were normalized to control and expressed as survival percentages. The IC_50_ was calculated after 72 h of GuaDex cells incubation.

### Cell cycle analysis

The analysis of the cell cycle was done by DNA content determination. Cells were plated in 6-well plates and incubated at 37° C for 24 h. GuaDex was incubated with the cells at 5 μM concentration for 24 h. The cells were harvested after incubation by a cell scraper and fixed in 4% formaldehyde in PBS at RT for at least 18 h. After fixation, cells were harvested and resuspended in 95% ethanol and kept at RT until analysis. Next, cells were washed with 2 ml dH_2_O, centrifuged, resuspended in 200 μL protease solution (0.1% Carlsberg’s solution Sigma protease XXIV in 0.1 M TRIS, 0.07 M NaCl, pH 7.2) and incubated at 37 °C water bath for 1 h. After incubation, 200 μL of 4′,6-diamidino-2-phenylindole (DAPI) at 8 μM solution (in 50 uM Sulphur-rhodamine 101, 1.1 M NaCl, pH 7.5) was added, and cells were analyzed on a BD LSRII (BD Biosciences, CA, USA) acquiring 10,000 events per sample. Flow cytometry data were analyzed using the ModFit LT software (version 3.2) (Verity Software House, USA).

### Actin detection assay

DAOY cells were seeded onto eight wells coverslips (Falcon, Corning, USA) for 24 h in media. The cells were then tested with 5 μM GuaDex compound for 1, 2, and 4 h in DMEM media and washed twice with PBS. Then, the cells were fixed in a 3.7% methanol-free formaldehyde solution in PBS for 30 min at RT and washed two times with PBS. Next, the cells were permeabilized with blocking buffer containing 0.1% TritonX-100 for 15 min, washed twice with PBS, and incubated overnight with 1% BSA in PBS at 4 °C. Next, the cells were incubated with primary antibodies 1:500 dilution in 1% BSA in PBS in a humidified chamber for 1 h at RT. Then, the cells were washed three times in PBS, 5 min, and incubated with 1 U/ml secondary antibody Alexa Fluor^®^ 488 phalloidin (ThermoFisher Scientific, Sweden) in 1% BSA in PBS for 1 h at RT. Next, the cells were washed thrice with PBS for 5 min in the dark. Finally, slides were prepared with a DAPI mounting medium (Vectashield, Immunkemi F&D AB, Järfalla, Sweden). A confocal microscope (Leica SP5, with a Leica Application Suite advance fluorescence 2011 Software, Leica Microsystems GmbH, Wetzlar, Germany) was used for image analysis.

### Tubulin detection assay

DAOY cells were seeded onto eight wells coverslips for 24 h in media. The cells were then incubated with GuaDex at 5 µM for 1 h in DMEM media and washed twice with PBS. Next, the cells were fixed in a 3.7% methanol-free formaldehyde solution in PBS for 15 min at RT and washed twice with PBS. Next, the cells were permeabilized with PBS containing 0.1% TritonX-100 at RT for 5 min, washed twice with PBS, and incubated overnight with 1% BSA at 7 °C. Then the cells were incubated with a 1:2000 dilution in a blocking buffer of primary mouse monoclonal β-tubulin antibody (Sigma-Aldrich, St. Louis MO, USA) for 1 h at RT. Next, the cells were washed with PBS three times and continued for 2 h at RT. After rinsing, the cells were 1 h incubated with a secondary mouse antibody 1:500 dilution Alexa Fluor^®^ 594 Anti-β Tubulin (ThermoFisher Scientific, Sweden) at RT. Finally, the cells were washed two times with PBS and prepared with DAPI mounting medium to label cell nuclei. A confocal microscope (Leica SP5) was used for the image analysis.

### Cell apoptosis detection

DAOY cells were seeded onto eight wells coverslips for 24 h in media. Rhodamine-GuaDex was prepared as previously [21]. The cells were PBS washed, incubated with Rhodamine-GuaDex at 5 µM for 1 h in PBS, and then washed twice with PBS. Cells were then incubated with 10 µL Annexin V-FITC detection kit (Sigma-Aldrich, St. Louis MO, USA) in PBS for 30 min at 4 °C dark and RT. After staining, the cells were washed carefully and prepared with DAPI mounting medium. A confocal microscope (Leica SP5) was used for the image analysis.

### Wound healing migration

A wound healing migration assay was carried out to determine the migratory ability of DAOY MB cells after being treated with GuaDex. The cells were seeded in 6-well plates (500,000 cells/well) in media until a monolayer formed for 24 h. Each well was manually scratched using a 200-μL pipette tip as previously described protocol [30]. The cells were washed three times with PBS. The cells were further incubated in media containing GuaDex at concentrations of 0.25 µM, 0.5 µM, and 1 µM. The control well was incubated with only media. After incubation, the cells were carefully washed with PBS. Images were obtained at the start and 24 h post-treatment using a phase-contrast microscope (Nikon Eclipse Ts2).

### Live-cell images of DAOY and GuaDex interaction

Study of the interaction dynamics of rhodamine labelled GuaDex incubated on DAOY cells, in vivo time-lapse analysis, confocal microscopy (Leica SP5). The cells in the media were seeded on a chambered coverslip with 4 wells and a #1.5H glass bottom (Ibidi GmbH, Graefelfing, Germany). Monolayer was formed after 24 h. Then the cells were washed two times with PBS, and DAPI was added plus 5 µM rhodamine-GuaDex in PBS buffer. The confocal microscope was implemented with a chamber system maintaining cell humidity at 37 °C and 5% CO_2_. Images were acquired at intervals of each 5 min for 80 min. Control DAOY cells were incubated with DAPI and 10 µl/ml propidium iodide in PBS, checking the viability duration of the cells. Magnification was 63x. Each image was pasted into a time-lapse analysis using the iMovie program (MacOS). Video dimensions were 640 × 360, color profile.H.264 Codec, SD (6-1-6).

### Statistical analysis

The experiments were conducted at least six times in all the experiments, except in the migration assay (three times). All data are expressed as the mean ± SD of at least three or six independent experiments. The significance level was set to p < 0.05.

The IC_50_ values (inhibitory concentration resulting in a 50% decrease in cell viability) were calculated from log concentration-effect curves using non-linear regression analysis in GraphPad Prism 7 for Mac OS X (version 7.0b (GraphPad Software, San Diego, CA, USA).

Cell cycle phase results were expressed as the percent average of cells in each cell cycle phase. Then two-way ANOVA was employed to analyze the statistical differences, followed by Tukey post-hoc analysis.

The cell migration was measured after the scratch assay, as described by Bobadilla et al. [31]. The distance migrated was monitored, and the area was measured manually by the area method. The area method assesses migration indirectly. During an experiment, the wound area percentage, *Â*(*t*), is tracked:

$$\hat A(t)=\hat A(t)\div A(0)\times 100$$

where *Â*(*t*) is the wound area at time *t* and *A*(0) is its initial area. The migration rate is then indirectly evaluated as the percentage of wound area at a specific time.

## Results

### Growth inhibition of GuaDex on medulloblastoma cells

**Fig. 1:**
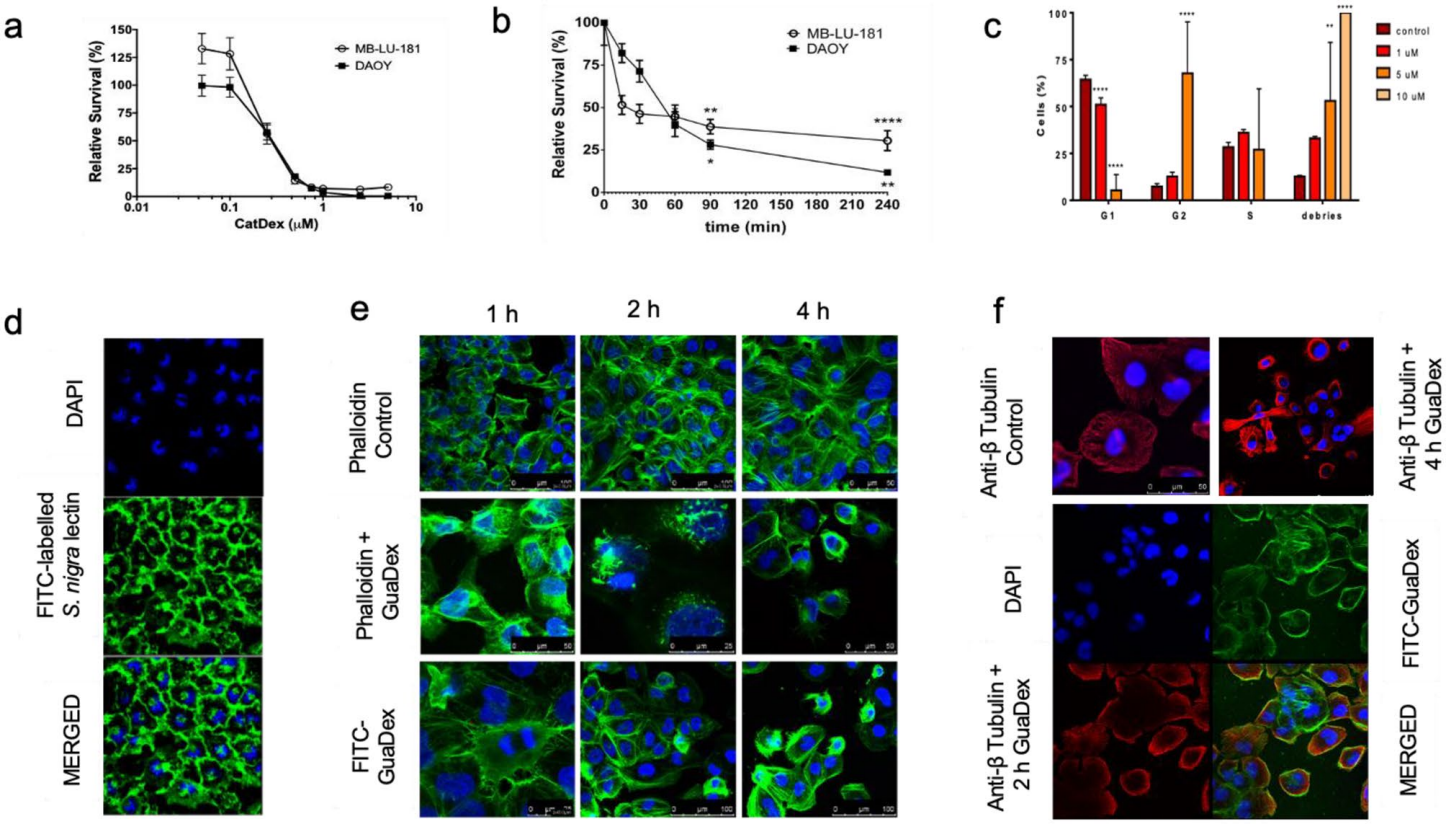
FMCA cytotoxicity assay: **a** DAOY and MB-LU-181 MB incubated 72 h with GuaDex at 0.5–8 µM concentration. **b** DAOY and MB-LU-181 MB cells incubated 4 h with GuaDex at 5 µM concentration. **c**. Flow cytometry: GuaDex concentration-dependent arrest of DAOY cells in G2/M cell cycle phase, 24 h incubation. Confocal microscopy: **d** DAOY cells, Sialic acid (Sia) expression, FITC labeled *S. nigra* lectin. **e** DAOY cells incubated for 1- 4 h with GuaDex at 5 µM concentration. Stabilization of actin filaments. **f** Stabilization of microtubule filaments at DAOY cells after incubation 2 and 4 h with GuaDex at 5 µM concentration. DAPI staining (blue), FITC-labeled *S. nigra* lectin (green) FITC-GuaDex (green), Alexa Fluor^®^ 488 phalloidin (green), Alexa Fluor^®^ 594 Anti-β Tubulin (red), Magnification 40x

Fluorometric cytotoxicity assay (FMCA) showed growth inhibition on DAOY and MB-LU-181 cells at 0.5 to 8 µM GuaDex after 72 h of incubation. The IC_50_ on DAOY was 223.4 nM, and on MB-LU-181 was 284.8 nM GuaDex after 72 h of incubation (Fig. 1a). The onset of cytotoxicity with 5 μM GuaDex concentration on MB-LU-181 MB cells was observed in less than 15 min and on DAOY in less than 240 min (Fig. 1b).

Flow cytometry was used to study the effect of GuaDex on cell cycle progression. GuaDex treatment altered mitotic spindles and induced G2/M phase cell cycle arrest leading to the inhibition of DAOY cell proliferation. After 24 h of GuaDex treatment, the flow cytometry analysis showed a marked decrease in the G1 cell cycle phase and an increase in the G2/M cell cycle phase (Fig. 1c).

FITC-labeled *S. nigra* lectin confirmed high expression of Sia on DAOY cell line (Fig. 1d).

### GuaDex induces changes in actin and microtubule networks and inhibits the migration of medulloblastoma cells

GuaDex effect on actin and microtubule**.** Alexa Fluor^®^ 488 Phalloidin and Alexa Fluor^®^ 594 Anti-β Tubulin immunodetection results showed that GuaDex when internalized, caused a change in the morphology of the microtubule network, polymerized actin and microtubule, and caused a rapid long polymerized bundles (Fig. 1e, f).

**Fig. 2:**
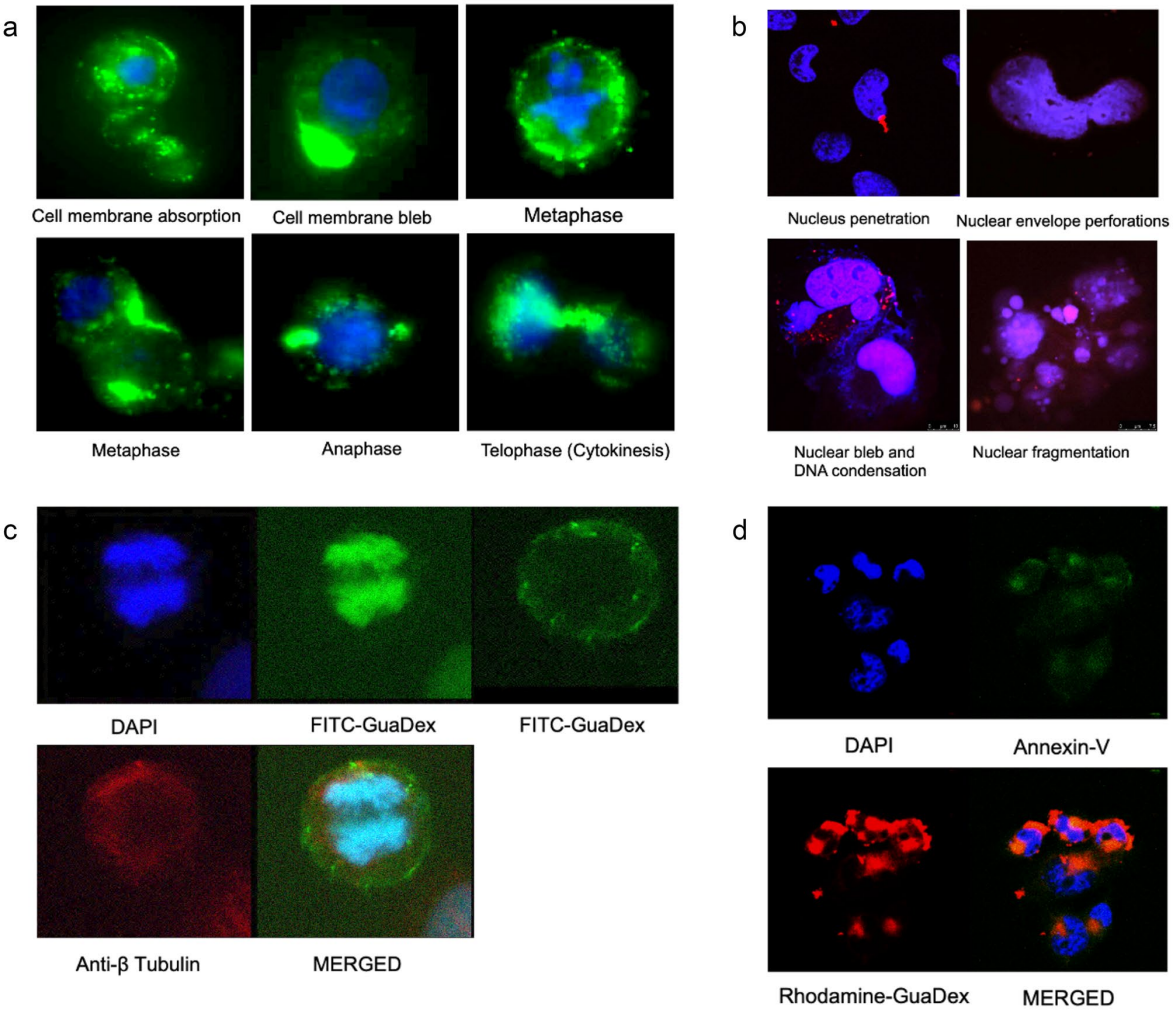
Fluorescent microscope images of DAOY cells. **a** 5 µM FITC-Guadex, 4 h incubation. FITC-Guadex cell membrane absorption, formation of blebs, binding to microtubule structures during cell cycle phases. DAPI (blue), and FITC-GuaDex (green). Magnification 63x. **b** Confocal microscopy images, nucleus, and DNA damage in DAOY cells incubated with Rhodamine-GuaDex at 5 µM concentration after 24 h incubation. Rhodamine-GuaDex (red) and DAPI nucleus staining (blue). Magnification 63x. **c** Immunofluorescent microscope images of DAOY cell in G2/M after 4 h incubation with FITC-GuaDex at 5 µM concentration. FITC-GuaDex (green) is binding with microtubule (red) and chromatin (blue). Magnification 60x. **d** Confocal microscope images. DAOY cells were incubated with Rhodamine-GuaDex (red) at 5 µM concentration for 1 h, Annexin-V assay (green). GuaDex appeared negative in the Annexin V apoptosis assay. Magnification 40x

Fluorescent microscope images. 5 µM FITC-Guadex 4 h treated DAOY cells. FITC-Guadex cell membrane absorption, no apoptotic blebs formation, and binding to microtubule structures during cell cycle phases (Fig. 2a). Rhodamine-GuaDex can penetrate the nucleus causing nuclear damage as, blebs, nuclear perforations, and nuclear fragmentation. Nuclear blebs facilitate the GuaDex binding to DNA and causing condensation, as shown in Fig. 2b. Guadex can bind microtubule and chromatin, promoting G2/M arrest (Fig. 2c). The GuaDex cytotoxic effect appears negative to Annexin V apoptosis assay. Figure 2d shows the FITC-Annexin V cell apoptosis analysis after 2 h, 5 µM Rhodamine-GuaDex treatment.

**Fig. 3:**
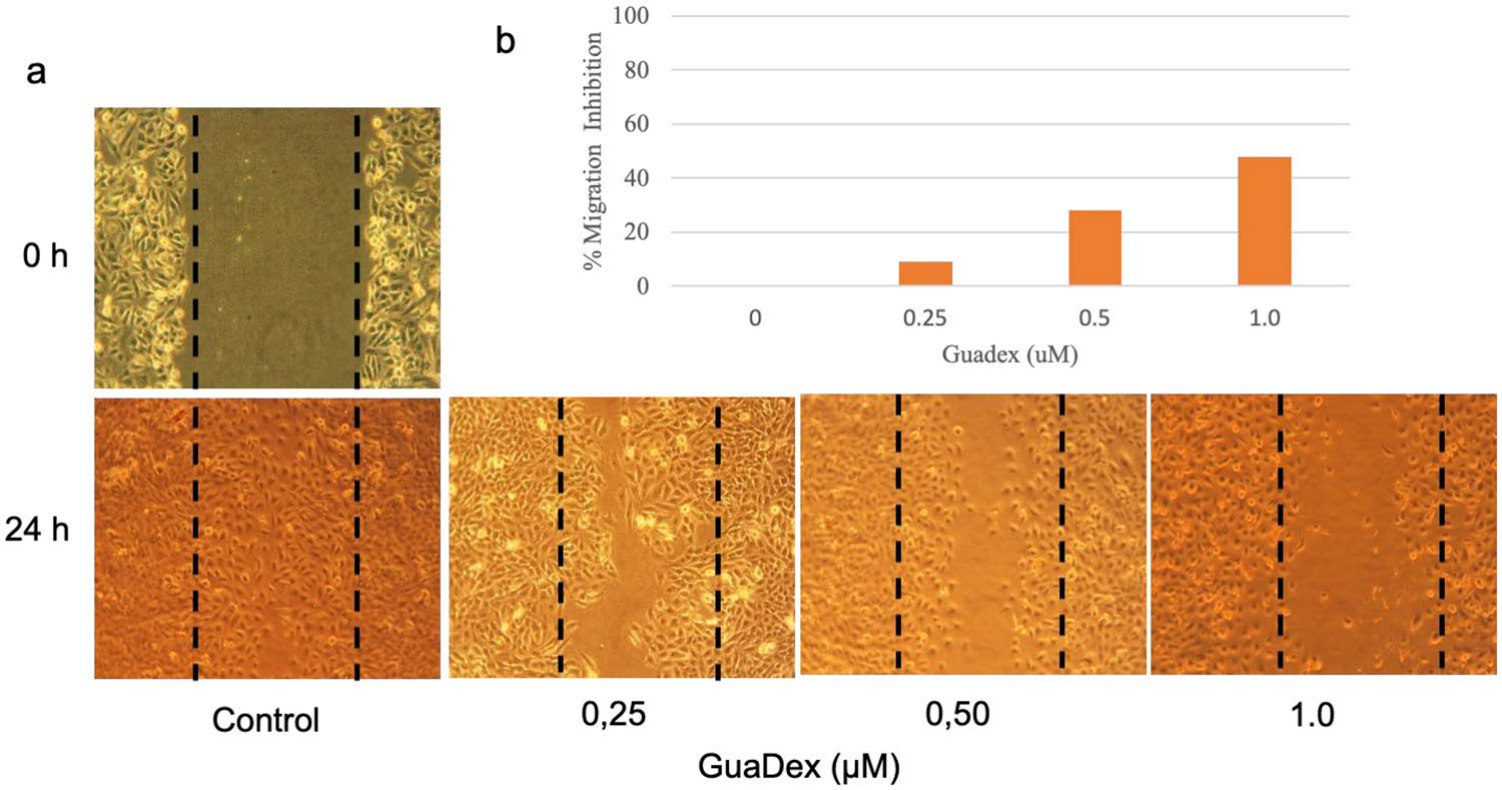
Guadex inhibitory growth effect on DAOY medulloblastoma cell lines after 24-h incubation at 0.25 to 1 µM. **a** Inverted microscope images, **b** Cell migration measured after the scratch assay, as described by Bobadilla et al. [31]. The distance migrated was monitored, and the area was measured manually by the area method. The cell migration rate was indirectly evaluated as the percentage of wound area at a specific time

GuaDex was found to significantly inhibit the migration of the DAOY MB in a concentration-dependent manner. The inhibition effect was observed at concentrations above 0.5 µM (inhibition of the DAOY confluent cell monolayer). The findings are based on the results of three independent experiments, and the data are presented as the mean ± SD. (Fig. 3a, b).

### Time-lapse analysis of Guadex-induced cytoskeleton disruption and cell denaturation in DAOY cells

The effect of Rhodamine-label GuaDex on DAOY cells through time-lapse analysis revealed significant cellular changes upon GuaDex binding to the cell membrane. Within the first five minutes, cytoskeleton disruption was observed, accompanied by the formation of no-apoptotic blebs on the cell membrane and blebs in the nuclear envelope (visualized through DAPI blue staining). After 15 min, there was an increase in cell volume, nuclear fragmentation, and nuclear perforations. DNA condensation was also observed, eventually increasing nuclear volume and cell denaturation after 35 min (captured in **video Daoy inc.GuaDex: m4v**).

The control cells’ video (description) showed DAPI staining of the cell nucleus after 35 min, followed by propidium iodide cell staining between 90 to 100 min later. Notably, the internalization of DAPI differs significantly between the control cells and the GuaDex treatment. GuaDex facilitates the internalization of DAPI, leading to pronounced staining and cellular changes within the observed time frame.

## Discussion

Targeted anticancer therapy aims to treat the tumor while sparing normal tissue and minimizing side effects. The success of this desirable intention depends on the specificity of the targeting entity and the exclusivity of the target that distinguishes it from normal tissue. In addition, several other factors affect the efficiency of targeted therapy, for example, the location of the tumor and its proximity to vital tissue, which is especially delicate in tumors of the CNS [32]. As a result, treatment of MB often causes severe complications due to side effects, particularly in pediatric patients with vulnerable growing brain [33].

Examples of treatment complications include cognitive impairment, dementia, psychiatric illness, bone growth retardation, hearing loss, and endocrine disruption [1].

MB presents divergent clonal selection at recurrence, and loss of targets at recurrence could contribute to a lack of success in treating recurrent disease. Metastatic spread results in death in almost all recurrent MB patients [34].

Guadex targets the cancer cells' aberrant glycosylation and hypersialylation (sia). Virtually all malignant tumors display aberrant cell surface glycosylation caused by altered metabolism.

In normal cells, glycans are essential for many biological processes, including cellular response to oxidative stress, resistance to innate immunity, intracellular protein trafficking, cell growth, and apoptosis [35]. Therapeutic antibodies and mucins with anticancer vaccine potential have emerged to diagnose and control glycosylation attempting to inhibit the invasive growth of malignant CNS tumors [36].

The cytoskeletal proteins, such as actin, tubulin, fascine, ezrin, and rho-GTPases, are becoming increasingly attractive targets for targeted therapy of brain tumors [37].

Abnormal actin and tubulin expression have been reported in many cancers, indicating that it may serve as an early cancer biomarker. For example, class β3-tubulin is a biomarker for neuroblastic central and peripheral nervous system tumors [38–40].

Different actin isoforms highlight mechanisms by which they may contribute to tumorigenicity. Moreover, the aberrant expression of the actin subunits can confer tumor cells with increased proliferation ability, migratory capability, and chemoresistance [10].

Breast cancer cell lines can mobilize the actin cytoskeleton in response to natural killer cell activity or by cytoskeleton remodeling, protecting against immune cell activity [41].

However, due to cardiotoxic side effects, many actin regulators have not been translated into clinical use [8].

Microtubule-targeting drugs are categorized into two groups: microtubule-stabilizing and destabilizing agents. The stabilizing agents, e.g., paclitaxel, docetaxel, epothilones, and discodermolide, bind to the tubulin polymer and stabilize the microtubules. On the other hand, the microtubule destabilizing agents, e.g., vinca alkaloids, colchicine, and combretastatin, bind to tubulin dimers and cause destabilization. These agents ultimately alter the equilibrium between tubulin and microtubule, disrupting the mitotic spindle [42].

Clinical use of microtubule inhibitors has significant side effects, e.g., neurological toxicity, bone marrow toxicity, and development of drug resistance [43].

GuaDex is a synthetic polymer conjugate comprising a carbohydrate backbone with multiple guanidine side groups, by definition, a polyamine. Polyamines are microtubule regulators through preferential electrostatic adsorption of their polyvalent cations to the anionic C-terminal tail of tubulin [44]. In addition, polyamines bind to DNA [45] through the highly anionic surfaces of nucleic acids [43]. The polyamines stabilize the DNA structure and, depending on their concentration and additional salt composition, induce DNA aggregation, often called condensation. However, the modes of interaction of these elongated polycations with DNA and how they promote condensation are still unclear [46]. Polyamines bind strongly to RNA, altering its structure sufficiently to change translation initiation sites and may affect the expression of proteins essential for cell migration. When inhibitors, mutation, or transfection reduce intracellular polyamines, severe reduction occurs in cell division, differentiation, and migration [47].

The analysis of GuaDex interaction with MB cells reveals its polyamine-like behavior and ability to denature tumor cells. GuaDex specifically targets the hypersialylation of the cell membrane. There was the formation of not apoptotic blebs when it internalized in the cytoplasm. This process involves the disruption of the actin cortex and the local membrane detachment from the cytoskeleton, as described by Paluch et al. 2013 [48]. GuaDex rapidly internalizes and binds to anionic intracellular structures such as actin, and tubulin, resulting in their polymerization.

Furthermore, GuaDex can penetrate the nucleus causing nuclear membrane damage as the formation of blebs. Like the detachment of the plasma membrane from the actin cortex, nuclear blebs involve the separation of the double membrane from the lamina. They may lead to the lamina shell's local rupture (s). This nuclear envelope rupture leads to an uncontrolled exchange between the nucleus and cytoplasm, resulting in DNA damage, as described by Shah et al. [49]. In addition, this nuclear envelope breach leads to the release of condensed DNA. The GuaDex intracellular electrostatic interactions ultimately contribute to the denaturation of the tumor cells, as shown in the image analysis.

The denaturation effect could be attributed to GuaDex size (m.w. ~ 55kD) and its high charge density, facilitating cross-linking and subsequent polymerization, leading to cell denaturation.

Group 3 of MB is characterized by the amplification of the Myc gene, which is associated with chemotherapy resistance. The JQ1 compound, the first identified BET inhibitor, downregulates Myc transcription [50]. In an orthotopic patient-derived MB-LU-181 medulloblastoma group 3 model, JQ1 showed an antitumorigenic response [25]. However, despite its antitumor activity, JQ1 presents limitations. It has a poor pharmacokinetic profile, low oral bioavailability, and a short half-life (1 h). Consequently, JQ1 must be administered twice daily to achieve a therapeutic effect. Although optimal dosing varies between tumor models [51]. BET inhibitors clinical trials for solid tumors showed a lack of significant responses, and adverse events are consistent [52].

GuaDex appears more cytotoxic on DAOY cells and the MB-LU-181 neurospheres with high stem cell content than previously reported resistance for chemotherapeutic drugs like cisplatin, temozolomide, and etoposide [24]. In a similar study, GuaDex showed high efficacy on glioma cell lines and glioma stem cell lines; however, MB cells appear even more sensitive with significant toxicity at lower concentrations [21]. Regarding side effects and what could be expected from GuaDex, a sister compound with a very similar structure has been investigated in phase I and phase II trials on castration-resistant prostate cancer patients (CRPC), demonstrating very mild side effects with no drug-related SAE (serious adverse events) recorded [53, 54].

However, the location of MB in the CNS makes it hard to predict side effects. In addition, the location of the blood–brain barrier makes the administration of GuaDex a problematic hurdle that remains to be resolved for possible future clinical studies.

## Conclusion

The poly-guanidine construct (GuaDex) shows potent cytotoxicity on MB cell cultures at low micro molar concentrations. Its mode of action involves extensive electrostatic cross-linking of crucial cell organelles, eventually causing cell denaturation. Further studies are warranted to explore the potential of GuaDex in the treatment of malignant CNS tumors. Particular attention should be given to the mode of administration and possible side effects, considering the delicate location of malignant CNS tumors.

### Supplementary Information

Below is the link to the electronic supplementary material.

Supplementary file1 (M4V 14 MB)

## Data Availability

The datasets generated during and analyzed during the current study are available from the corresponding author upon reasonable request.
